# Enabling recruitment success in bariatric surgical trials: pilot phase of the By-Band-Sleeve study

**DOI:** 10.1038/ijo.2017.153

**Published:** 2017-08-15

**Authors:** S Paramasivan, C A Rogers, R Welbourn, J P Byrne, N Salter, D Mahon, H Noble, J Kelly, G Mazza, P Whybrow, R C Andrews, C Wilson, J M Blazeby, J L Donovan

**Affiliations:** 1 School of Social and Community Medicine, University of Bristol, Bristol, UK; 2 Clinical Trials and Evaluation Unit, School of Clinical Sciences, University of Bristol, Bristol, UK; 3 Department of Bariatric and Upper Gastrointestinal Surgery, Musgrove Park Hospital, Taunton, UK; 4 University Surgery Unit, University Hospitals Southampton, Southampton, UK; 5 NIHR CLAHRC West (National Institute for Health Research Collaboration for Leadership in Applied Health Research and Care West), University Hospitals Bristol NHS Trust, Bristol, UK

## Abstract

**Background:**

Randomized controlled trials (RCTs) involving surgical procedures are challenging for recruitment and infrequent in the specialty of bariatrics. The pilot phase of the By-Band-Sleeve study (gastric bypass versus gastric band versus sleeve gastrectomy) provided the opportunity for an investigation of recruitment using a qualitative research integrated in trials (QuinteT) recruitment intervention (QRI).

**Patients/Methods:**

The QRI investigated recruitment in two centers in the pilot phase comparing bypass and banding, through the analysis of 12 in-depth staff interviews, 84 audio recordings of patient consultations, 19 non-participant observations of consultations and patient screening data. QRI findings were developed into a plan of action and fed back to centers to improve information provision and recruitment organization.

**Results:**

Recruitment proved to be extremely difficult with only two patients recruited during the first 2 months. The pivotal issue in Center A was that an effective and established clinical service could not easily adapt to the needs of the RCT. There was little scope to present RCT details or ensure efficient eligibility assessment, and recruiters struggled to convey equipoise. Following presentation of QRI findings, recruitment in Center A increased from 9% in the first 2 months (2/22) to 40% (26/65) in the 4 months thereafter. Center B, commencing recruitment 3 months after Center A, learnt from the emerging issues in Center A and set up a special clinic for trial recruitment. The trial successfully completed pilot recruitment and progressed to the main phase across 11 centers.

**Conclusions:**

The QRI identified key issues that enabled the integration of the trial into the clinical setting. This contributed to successful recruitment in the By-Band-Sleeve trial—currently the largest in bariatric practice—and offers opportunities to optimize recruitment in other trials in bariatrics.

## Introduction

Randomized controlled trials (RCTs) including surgical procedures have long been acknowledged to be difficult to mount because of methodological, cultural, historical and technical issues.^1, 2, 3, 4^ Hence, there are fewer RCTs than in other clinical specialties,^5, 6, 7, 8^ meaning that only a small proportion of surgical treatments are based on the highest level of evidence generated from RCTs.^9^ In addition, there has been a lack of exposure to and experience of RCT research in many surgical specialties,^10^ although this is now changing with initiatives such as the Royal College of Surgeons Surgical Trials Centers^11^ and the surgical trainee-led research collaboratives.^12^

The By-Band-Sleeve RCT (ISRCTN: 00786323) was initially established to compare gastric bypass and gastric banding for complex obesity^13^ and it adapted to a three group trial to also compare sleeve gastrectomy in the main phase.^14^ These operations were in common use but lacked robust comparative evidence and longer-term follow-up data, with observational studies suggesting that the different techniques led to similar long-term weight loss but different profiles of weight loss. The ongoing trial aims to recruit a total of 1341 patients to compare the three operations with respect to weight loss and health-related quality of life 3 years after randomization.

This RCT was anticipated to be challenging as the bariatric surgical community within the United Kingdom and internationally was known to be divided in its preference for and expertise in the operations. Similarly, the nature of the condition also meant that patients had often been considering surgery for an extended period of time, which was felt to lead to the development of entrenched views partly influenced by patients' local support and advocacy groups. A qualitative research integrated in trials (QuinteT) recruitment intervention (QRI),^15^ as developed originally in the ProtecT study^16, 17^ and subsequently refined and applied to a number of completed^18, 19, 20, 21^ and ongoing RCTs, was therefore integrated into the RCT at its inception to investigate, identify and address recruitment problems. The QRI is the key component of an extensive program of research aimed at optimizing recruitment and informed consent in surgical RCTs as part of the Medical Research Council's ConDuCT-II (Collaboration and innovation in Difficult and Complex randomized controlled Trials In Invasive procedures) hub, Royal College of Surgeons Bristol Surgical Trials Center and the QuinteT research team.

This article reports how the QRI improved recruitment in the pilot phase of the By-Band-Sleeve RCT (which recruited into two groups, gastric bypass and gastric band) so that it proceeded to the main trial and prepared the way for the inclusion of new clinical centers.

## Materials and methods

The QRI was integrated^22^ within the By-Band-Sleeve RCT protocol and carried out in the two pilot participating centers (Center A and Center B) in two iterative phases.^15, 16, 17, 18, 19^ Ethical approval was obtained from the National Research Ethics Service Committee South West—Frenchay (reference number: 11/SW/0248). Informed consent was sought from staff and patients before non-participant observation and audio recordings of appointments and interviews.

### Understanding recruitment

In order to thoroughly comprehend the recruitment process, all components of the QRI^15^ were carried out in this study (see Tables 1 and 2 for details):

In-depth semistructured interviews (*n*=12) were conducted by SP before the commencement of recruitment with a purposive sample of staff members involved in the trial design, eligibility assessment, information provision or informed consent processes.^15^ Interviews aimed to identify ‘clear obstacles’ and ‘hidden challenges’ to recruitment.^23^ All interviews were transcribed verbatim and analyzed thematically using constant comparative methods^24, 25^ to identify the similarities and differences across participants, centers and disciplines. A coding frame was established (SP and JLD) with emerging themes iteratively explored in new interviews. A descriptive account (SP) of the themes was written up with charts showing patient pathways through recruitment.

Audio recordings (*n*=84) were routinely made of all appointments between recruiters and potentially eligible patients. Overall, the majority of those available were analyzed (84/90; Table 1). Discussions about the surgical options for weight loss and aspects of the By-Band-Sleeve study were transcribed. All recordings were repeatedly listened to, with detailed notes made on the interaction, and analyzed together (JLD and SP). Targeted conversation analysis^16^ focused on aspects such as how recruiters demonstrated equipoise, explained the two operations, the trial and randomization, with the aim of elucidating 'hidden challenges.^23^

Non-participant observations^26^ (*n*=19) of consecutive patients' appointments with clinical staff was carried out during the first three clinic days in Center A (15 appointments) and the first two clinic days in Center B (4 appointments) (SP) to provide preliminary understanding of the clinical setting and early recruitment. In Center A, an education session (where groups of patients were given information on their surgical options, diet and the RCT) was also observed. Detailed field notes were made to document the organizational structure of the clinics and staff-patient interaction; notes were thematically analyzed as interviews above.

Eligibility and recruitment logs routinely captured detailed information on the number of patients who were screened, eligible, approached and randomized (SEAR framework^15^). The data were scrutinized (trial team, JLD and SP) to monitor pre- and post-feedback recruitment rates and trends in treatment preference among those who declined randomization.

### Plan of action

The data from all of the above aspects of the QRI were analyzed rapidly and a plan of action was developed collaboratively with the chief investigator (CI), center recruiters, clinical trials unit and the QRI team, to address the recruitment problems identified by the QRI. Group and individual feedback sessions were organized at each center to present anonymized findings in a confidential and supportive manner.

The implementation of the plan of action was monitored and evaluated through: (a) analysis of post-feedback audio recordings and regular telephone discussions with center staff and (b) monthly reports on the eligibility/recruitment logs—to track changes in post-feedback randomization rates. The plan of action flexibly accommodated emerging findings.

The key findings from the QRI in the two clinical centers over the first 14-month period of the pilot phase of the RCT are presented below.

## Results

### Understanding recruitment in Center A

**Figure 1:**
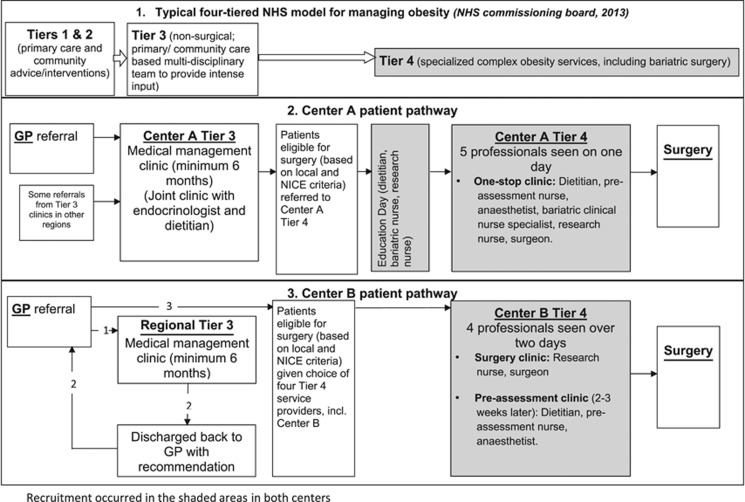
Patient pathway across Centers A and B.

Center A had long-standing routines for clinical service provision that followed the recommended four-tiered patient pathway,^27^ with Tier 4 involving bariatric surgery (Figure 1). Patients were first given an overview of the RCT and the patient information leaflet during an education session and detailed information was provided in the One-Stop Clinic, where they met five professionals in one afternoon. Surgeons were the primary study information providers, with support from research nurses. The two key recruitment challenges in this center and the plan of action are presented below.

#### Integration of the trial into clinical practice

The key recruitment challenge in Center A was that their well-established routines for clinical service provision led to the trial being presented to patients as an ‘add-on’ extra rather than an integral part of existing clinical services. This had an effect on what was said about the study and the eligibility assessment. First, recruiters did not change their consultations and so the study was first mentioned only toward the end of the appointment. It was briefly discussed, usually in an apologetic tone and only as an option for patients who did not have a *‘strong preference’* (Table 3). This gave the impression of the trial being a digression from usual practice rather than a proposition that warranted deliberation. Study concepts such as the rationale for the study and randomization were not clearly explained or linked. For example, although recruiters often mentioned they did not know which treatment was best, they did not clearly link uncertainty to the rationale for the study. This meant that although patients became uncertain about what treatment to have, study participation was not explicitly offered as a solution to this uncertainty. In addition, follow-up provided in the study was not presented within the context of NHS follow-up provision but as an additional burden for the patient.

Second, the pathway for patients through eligibility assessment and then randomization was complex and unclear. Potentially eligible patients were given study information during the One-Stop Clinic, but final confirmation of their eligibility was determined during the multi-disciplinary meeting at the end of this clinic or at a later date following clinical tests. This meant that it was often not possible to randomize eligible patients who were willing to participate in the study on the clinic visit day. As a result, randomization had to occur on another day once tests had been completed, and some patients were subsequently lost to the study. Furthermore, surgeons who tended to see patients after other staff, did not discuss the study if other staff had noted that the patient had a preference for one operation.

#### Recruiter equipoise and addressing patient preferences

Recruiters’ conflicting views about equipoise were a key hidden challenge^23, 28^ to recruitment. In interviews, recruiters discussed their attempts to overcome their treatment biases (mostly in favor of gastric bypass) to achieve equipoise in light of the merits of the study. However, in consultations, they found it difficult to maintain equipoise and patients tended to decline study participation, often choosing bypass surgery. There was evidence from audio recordings that the terminology used by recruiters in the appointments favored bypass and they tended to present it more positively than band surgery (Table 4).

Recruiters provided detailed information on each procedure. Some recruiters were familiar with the literature^16, 17, 18, 19, 29^ emphasizing the importance of exploring patient preferences. However, there was evidence from the recordings that recruiters asked patients ‘why’ they had a stated preference, but then tended to accept the reason offered at face value (Table 3). The treatments were referred to as *‘permanent’* (bypass) or *‘temporary’* (band) solutions, and although surgeons acknowledged in interviews that both procedures were designed to be permanent, these views were not countered in the appointments (see Table 4 for reasons mentioned by patients for preferences). Similarly, recruiters were aware of advice in previous publications^17^ regarding balanced information provision on the two operations, but they often did not tailor or balance the information in relation to patients’ concerns.

### Plan of action

Individual feedback was provided to two surgeons and the research nurse 2 months after recruitment commenced and a group feedback meeting was held in the third month to discuss these issues further. In order to ensure that all professionals regarded the study as an integral part of clinical service provision, non-recruiters (anesthetists, pre-assessment nurses, dietitians) and recruiters (surgeons and research nurses) were invited to the feedback session. A series of recommendations to improve recruitment was made and discussed (Table 5).

Although most suggestions were taken on board, (see Table 3 for comparison of before and after feedback quotes), there were some persistently problematic issues (for example, the lack of a clear randomization explanation) and some new issues, which were presented at subsequent feedback sessions.

Center A showed more than a fourfold increase in recruitment, from 9% (2/22) in the first 2 months of recruitment to 40% (26/65) in the next 4 months after feedback sessions. Over the next year, various other feedback sessions were conducted and a ‘tips’ document was circulated, and recruitment stabilized at about 38%.

### Center B

At the time of the By-Band-Sleeve study being planned, Center B was going through a process of restructuring. Patients from Tier 3 were discharged back to Tier 2, where they were given a choice of Tier 4 service providers, including Center B (Figure 1). Tier 4 obesity surgery provision was spread across the private and NHS sectors, leading to delays in commencing recruitment because of research governance issues (Center B began 3 months after Center A). The unintended staggered start in recruitment between the two centers meant that the lessons learnt from Center A trickled through to Center B through presentations at the Trial Management Group meetings and other informal means. Center B established a pre-assessment clinic day (anesthetist, dietitian and bariatric nurse) separately from the consultation with the surgeon and the research nurse (unlike the single clinic in Center A). The staff in Center B were able to spend considerably longer presenting the study and used a balanced format to describe the two operations. These factors contributed to a promising start in recruitment at Center B, with 39% (9/23) of eligible patients agreeing to be randomized in the first 5 months of recruitment.

Although the RCT was well integrated into clinical practice from the start, the most important challenge in Center B was in relation to recruiter equipoise. In interviews, recruiters stated that they were aware of their biases and were careful not to convey that to patients. However, in consultations with patients, they sometimes expressed a preference for a particular operation for specific groups of patients (undermining equipoise) or made an explicit treatment recommendation (overriding equipoise) without discussing the By-Band-Sleeve study.^30^

### Plan of action

Suggestions similar to Center A were provided in relation to equipoise and dealing with patient preferences. The center consistently implemented the recommended strategies and following feedback, their recruitment stabilized at about 45% over the next year.

The By-Band-Sleeve study successfully completed pilot recruitment and progressed to the main phase with a total of 11 centers. The QRI is currently ongoing across all the centers.

## Discussion

A QRI was included in the pilot phase of the By-Band-Sleeve study because recruitment to the trial was expected to be challenging. In Center A, the QRI rapidly identified that the center’s effective routines for clinical service provision did not facilitate trial recruitment: the trial needed to be better integrated into existing processes. The plan of action to address this and the other key issues related to equipoise and treatment preferences was well implemented by the center, and recruitment increased markedly. Center B’s delayed start and the need to institute a new clinic meant that it was easier to integrate the trial processes into the clinical service. This led to a good start to recruitment and support was targeted to issues related to equipoise and preferences. The QRI was able to identify key difficulties with recruitment that could be addressed in these two centers and contributed to the successful progress from the pilot phase to the main phase. Consideration should be given to including a QRI intervention into future surgical trials where challenges with recruitment are expected.

The importance of integrating the trial into clinical services^31^ to ensure a seamless service provision for patients that also aided trial recruitment was a pivotal finding highlighted by the QRI in Center A. Viewing the trial as a discrete entity from clinical service meant it was not given sufficient consideration by recruiters or patients. Time spent on discussing the RCT was not quantified in the By-Band-Sleeve study, but limited discussion of the RCT has previously been reported to be an important barrier in other studies.^32^ Similarly, being apologetic about offering the study, unclear explanations of RCT concepts such as randomization and, consequently, patients’ poor understanding of the trial and its underlying concepts have also been previously documented.^16, 17, 20, 23, 28^ In the By-Band-Sleeve study, we also noticed a lack of appropriate links made between recruiters’ statements of uncertainty about which treatment was best and the rationale for the study. Such missing links across various aspects of trial information provision may need further exploration in other RCTs.

Problems arising from complex patient pathways^19, 21, 23^ have previously been documented. Although it is likely that these pathways are rarely straightforward, surgical RCTs have an added layer of complexity because of the need for consensus across professionals from multiple disciplines that the patient indeed needs a major operation. In this study, in addition to the surgeon, the endorsement of the anesthetists, dietitians and bariatric clinical nurse specialists was necessary for surgery and their views played a crucial role in the decision-making process of the patients. The trial benefitted by including all these staff in all aspects of the QRI, including feedback, so that they were committed to the trial. Also, the disconnect between the study processes and existing clinical service provision meant there was confusion over when a patient was deemed fully eligible and when an eligible patient willing to participate in the study could be randomized. This finding is likely to resonate with many RCTs as most will need patients to go through a series of tests to confirm eligibility and without clear strategies and pathways in place, there may be uncertainty about the time of randomization amongst recruiters.^19^

It has recently been found that clinicians working in trials that compare treatments from different disciplines tend to favor the treatment routinely offered by their own specialty.^33^ In By-Band-Sleeve, the procedures offered were from just one specialty, bariatric surgery, and yet it appeared that recruiters displayed an unintentional tendency to favor the more routine procedure, usually bypass, which was carried out in their center before the trial.

In Center B, the QRI influenced recruitment through the transfer of lessons learnt from Center A, specifically in relation to the set-up of a clinical structure that enabled recruitment. Other issues around surgeons’ equipoise in relation to patients who stood at the periphery of the formal eligibility criteria and their tendency to unwittingly or explicitly communicate their own preferences to the patient have previously been documented^19, 21, 23, 28, 34^ and were addressed in feedback sessions.

Lessons learned from the QRI during the pilot phase were incorporated into the pre-recruitment training provided during site initiation visits in the nine new centers. In order to responsively provide solutions to site-specific challenges, the QRI is currently ongoing across all the 11 centers. The QRI has involved an ongoing process of monitoring recruitment information, analyzing audio recordings and providing regular feedback to each of the 11 sites to discuss issues as they arise to support surgeons and research nurses in discussing trial concepts and providing balanced, patient-focused information. This iterative process means that By-Band-Sleeve is continuing to recruit successfully in its main phase despite being a complex multi-centered surgical trial.

The primary strength of the QRI described in this article is in its methods, developed,^16, 17^ employed and fine-tuned over multiple applications in RCTs that span a range of conditions and disciplines, including both purely surgical comparisons, such as the RCT reported in this article, surgical versus non-surgical comparisons and trials comparing other health technologies,^18, 19, 20^ with capacity to adapt and introduce innovative methods as necessary for the RCT.^32^ The iterative and collaborative nature of the intervention, triangulation through the use of multiple data sources to ensure that the findings were grounded in the data and rapid feedback that is mindful of the tight timeframes faced by RCTs were all key to precipitate changes in practice amongst recruiters. The QRI in this study experimented with the addition of non-participant observations of consultations to gain a rapid insight into the recruitment pathway/practices.

Another crucial strength of the QRI lay in its ability to identify clear obstacles and hidden challenges drawing from findings in other RCTs,^23^ while also identifying the recruitment challenges exclusive to each—such as surgeons’ demotivation if a patient was indicated to have a preference for a particular operation by other professionals. The need for a tailored QRI was evident from the findings that previous published work on patient preferences^16, 17, 19, 20, 21, 23, 28, 29^ and balancing of information^16, 17^ were misinterpreted by recruiters and required further training and support. In addition, continuous monitoring of recruitment practices is crucial to identify emerging issues, such as the finding that patients who were seen earlier in the clinic appeared to be more likely to be randomized. Therefore, while generic training for recruiters drawing from previous QRI findings would be of benefit,^35^ the above findings reinforce the need for RCT-specific QRIs to identify and plan for the unique challenges in each RCT.

The main limitation of the QRI reported here was the difficulty in directly attributing recruitment success in the By-Band-Sleeve study to the QRI alone—recruitment success was also the result of the hard work and commitment of the clinical and clinical trials unit staff. Although the findings were only based on the two centers participating in the pilot phase, this meant that recruitment could be explored in depth to inform the main phase. Previous limitations of the QRI such as the lack of adequate number of audio recordings^18, 19^ were addressed by integrating the QRI into the trial (for example, processes for audio recording of consultations were included in the trial manual by the clinical trials unit rather than provided separately). This enabled a broader understanding of the key issues that could be presented with credibility at feedback sessions.

## Conclusion

The QRI reported here demonstrates the importance of early identification and addressing of recruitment challenges, and the need for extending the QRI throughout the recruitment period of surgical RCTs that may be difficult to recruit into to sustain benefits gained. The most important contribution of the QRI in the By-Band-Sleeve study was in identifying key issues rapidly, including highlighting the importance of integrating an RCT into a center’s clinical provision, in addition to addressing other challenges related to equipoise and treatment preferences. Integrating the QRI in the pilot phase of this trial demonstrated to the funders of the trial that recruitment could be improved from a poor start, and provided confidence in extending recruitment to other bariatric centers in the main trial. The By-Band-Sleeve trial, currently the largest in bariatric practice, continues to recruit from 11 centers (*n*=789 randomized by June 2017) and it is expected to complete recruitment in 2019. The QRI continues to provide training and support to ensure that recruitment is optimized and patients make an informed choice regarding participation in the By-Band-Sleeve study. The QRI in this article demonstrates that there are opportunities to optimize recruitment in other trials in bariatrics.

## Data availability

The data (transcripts) that support the findings of this study are available on request from the corresponding author. The data are not publicly available because of them containing information that could compromise privacy/consent, but the authors will be able to consider specific requests on a case-by-case basis.

## Members of the By-Band-Sleeve TMG

The By-Band-Sleeve TMG members also include Barney Reeves, Sarah Wordsworth, Janice Thompson and Mary Perkins.

## Figures and Tables

**Table 1:** QRI sample, data set and feedback sessions

*Sample and data collection*	*Center A,* n		*Center B,* n		*Trial management group members,* n	*Total*
*Staff interviews*						
Sample: everyone involved in patient pathway and/or trial design	5 (2 surgeons, 1 endocrinologist, 1 bariatric nurse, 1 dietitian)		6 (3 surgeons, 2 pre-assessment nurses, 1 dietitian)		7 (1 surgeon-CI, 1 study lead each for nutrition, health economics, methodology, statistics, epidemiology, patient/public involvement)	18
Interviewed	4 (all of sample except dietitian)		6 (all of sample)		2 (CI and nutrition lead)	12
*Audio recordings of appointments*^a^	Feedback 1	Feedback 2	Feedback 1	Feedback 2^b^	—	
Sample: recordings available	28	19	24	36		90
Recordings analyzed	22	19	24	19		84^a^
*Non-participant observations*						
Appointments	15	—	4			19
Education day	1	—	—	—	—	1
Feedback sessions	1 Group 3 Individuals	1 Group 2 Individuals	1 Group 3 Individuals	— 2 Individuals	—	3 Groups 10 Individuals

Abbreviations: CI, chief investigator; QRI, qualitative research integrated in trials (QuinteT) recruitment intervention.

a Numbers provided are of patients. Each patient saw 2–5 health-care professionals; total number of recordings analyzed was therefore upward of 168 (84 × 2).

b Center B’s second feedback session only involved individual feedback to two recruiters as the center was recruiting well; one new research nurse (*n*=12) and one surgeon, who had particular concerns regarding consent rates/withdrawals in two specific clinics (*n*=7), and all the relevant recordings were analyzed (*n*=19).

**Table 2:** Staff participants’ profile

*Staff ID*	*Center*	*Role*^a^	*Data set contribution*		
			*Interviews*	*Observations*	*Audio recordings*
S01	Center A	Recruiter	✓	✓	✓
S02	Center A	Recruiter	✓	✓	✓
S03	Center A	Non-recruiter	✓	x	x
S04	Center A	Non-recruiter	✓	✓	✓
S05	Center A	Recruiter	x	✓	✓
S06	Center A	Non-recruiter	x	✓	✓
S07	Center A	Recruiter	x	✓	✓
S08	Center B	Recruiter	✓	✓	✓
S09	Center B	Recruiter	✓	x	✓
S10	Center B	Recruiter	✓	x	x
S11	Center B	Non-recruiter	✓	x	x
S12	Center B	Non-recruiter	✓	x	x
S13	Center B	Non-recruiter	✓	x	x
S14	Center B	Recruiter	x	✓	✓
S15	Center B	Recruiter	x	x	✓
S16	TMG	Non-recruiter	✓	x	x
S17	TMG	Non-recruiter	✓	x	x
Total	Center A: 7 Center B: 8 TMG: 2	Surgeons: 7 Research nurses: 3 Endocrinologist: 1 bariatric nurse: 1 Dietitian: 2 Pre-assessment nurse: 2 Nutritionist: 1 ------ Recruiters: 9 Non-recruiters: 8	12	8	10

Abbreviation: TMG, Trial Management Group.

a Clinical roles, such as surgeon, endocrinologist, research nurse and dietitian, are not provided as it is likely to identify the participant.

**Table 3:** Comparison of recruiter quotes from consultations before and after feedback: tracking the changes in one Center A recruiter, S02

*1. Study presentation*
A. Before feedback
S02: Now at the moment we're in a situation where we know they both work, they both help people to lose weight and they both help people to improve their life. We don't know which one of those is the best really. Some people have particular preferences for one operation or the other, but for people that don't have any particular preferences, we're running this trial. So hmm have you got, you know cast iron preferences? (Audio recording of consultation, Center A, surgeon).
B. After feedback
S02: We're currently doing a study to try and find out if one is better than the other or if they're equivalent, 'cause you know, in the past we've had lots of people choose one, and lots of people choose another, often for no particular reason, and they've both tended to do well. So there's lots of arguments in the community now, I think this one's better and the other guy says I think this one's better, nobody really knows (Audio recording of consultation, Center A, surgeon).
*2. Eliciting, exploring and addressing preferences and balancing of information*
A. Before feedback
Patient: I would prefer the gastric bypass. S02: Okay, what is it that makes you prefer that operation? (Audio recording of consultation, Center A, Surgeon). Patient: I think once it's done, it's done. You lose the weight quicker to begin with, but I know it equals out... to me that seems more what I want because with the band your to-ing and fro-ing, I've got a 9 year old to look after.
S02: Okay that's fine. I just want to let you know what's available and if you've got a particular preference then that's fine.
B. After feedback
Patient: I personally think that a bypass would do me... S02: Right, what is it that makes you think that? (Audio recording of consultation, Center A, Surgeon). Patient: Well, once the job's done, it's done, there's no turning back unless there's an emergency right, but I think to myself that I just wanna get it done and over with, especially with the haemophilia. I want nothing rubbing or moving inside to create bleeding. S02: Well, the band should be considered as permanent as well, once it's in, it's in, and it shouldn't rub and cause any bleeding or anything. We wouldn't expect that (laughs lightly), if we would, we wouldn't put it in anyone who had haemophilia. Patient: Yeah, well the other thing is you're taking injections as well after that, aren't you? S02: After bypass you'll have to have vitamin B12 injections. With the band you have to have injections into the band, so both of them involve injections.

**Table 4:** Links between terminology used by recruiters and reasons mentioned by patients for preferences

*Terminology used by recruiters to describe*	*Reasons mentioned by patients for preferences*	
	*For*	*Against*
*Bypass*^a^		
• Guaranteed and gets you/carries you there	- Permanent	- Too permanent
• Once it’s done, it’s done	- Once it’s done, it’s done	- Too rapid weight loss
• Restriction ‘and’ malabsorption	- Rapid weight loss	- Too severe/complicated operation/strong anesthetic, stomach cut
• Not much else you can do after bypass	- Straight-forward	- Needle phobia (bypass needs lifelong vitamin injections)
• 60–70% excess weight loss	- Prevents cheating	- Only for heaviest
		- Dumping syndrome
		- Excess skin
		
*Band*^b^		
• Requires a lot more patient input/is hard work and you have to get there yourself	- Only temporary/can be removed/can be adjusted	- Not permanent
• You can cheat on the band	- Less rapid weight loss	- Slow/not enough weight loss
• Only restriction	- Easier operation	- Can cheat
• You still have options post band	- Weaker anesthetic	- Foreign object inside
• 40–50% excess weight loss	- Less excess skin	- Fiddly follow-up
		- Needle phobia (band adjustments with a chest needle)

a Information in the patient information leaflet (PIL) regarding bypass surgery: entails an operation that takes 1 to 2 h in which a small pouch is made in the top of the stomach and a loop of bowel connected to this pouch to bypass the rest of the stomach. A 1- to 3 -day hospital stay is required. Follow-up schedule of clinic visits are required at 4 weeks, 3, 6 and 12 months after surgery and annually thereafter. Patients are required to take long-term vitamin and mineral tablets and also require regular 3 monthly vitamin B12 injections. Possible advantages/disadvantages include that it requires significant dietary modification by the patient; rapid weight loss in first 6 months, which slows then stabilizes at 18 months; at this stage weight may be regained and can be significant in about one in five patients; and if significant weight loss is achieved, health problems such as diabetes may resolve.

b Information in the PIL regarding band surgery: entails an operation that takes less than 1 h in which a band is inserted around the top of the stomach to reduce its size. An overnight stay in hospital is usually required. Follow-up schedule of clinic visits for band adjustments are required. There may be up to 10 visits in the first 2 years. Patients are required to take long-term vitamin and mineral tablets. Possible advantages/disadvantages include that it requires significant dietary modification by the patient; weight loss is gradual over a long period (2–3 years); and if significant weight loss is achieved, health problems such as diabetes may resolve.

**Table 5:** Center A—recruitment challenges and corresponding QRI solutions

*Recruitment challenges*	*QRI suggestions/actions*
Trial not well integrated into clinical practice	• Mention the study in the opening statements of the surgical consultations. • Offer the study to ‘all’ eligible patients. • Provide detailed and consistent messages across professionals in relation to concepts such as randomization. • Link concepts such as uncertainty (not knowing which is best) to the study rationale. • Present trial follow-up within the context of usual NHS follow-up. • Plan pathway for potentially eligible patients who were willing to be randomized but needed further tests to confirm eligibility. • Do not indicate patient preference anywhere on the notes (for staff members who saw patients before the surgeon). • Express enthusiasm for the study.
Recruiters’ equipoise dilemmas and addressing patient preferences	• Feedback sessions used to make recruiters aware of instances where they inadvertently used loaded terminology (Table 4) or conveyed their own preferences to patients. • Move beyond initial probing questions in relation to patient preferences toward rectifying any erroneous views (there was consensus that barring a few reasons given by patients for their treatment preferences, most were not based on evidence or were factually incorrect; it was agreed that this would be conveyed to patients to ensure that patients had been fully informed in their decision making). • Request patients who appear to arrive at the clinic with a preference or decision about trial participation to ‘keep an open mind’ until they had heard all the relevant information. • Balance the operations in relation to the patient's concerns rather than just each other (for example, many patients objected to the gastric band as it meant there was a 'foreign body' inside them; it was important to therefore let them know that the gastric bypass also involved staples, which were left inside).

Abbreviation: QRI, qualitative research integrated in trials (QuinteT) recruitment intervention.
